# Modeling Neurological Disorders in 3D Organoids Using Human-Derived Pluripotent Stem Cells

**DOI:** 10.3389/fcell.2021.640212

**Published:** 2021-05-10

**Authors:** Raj Bose, Soumyabrata Banerjee, Gary L. Dunbar

**Affiliations:** ^1^Field Neurosciences Institute Laboratory for Restorative Neurology, Central Michigan University, Mount Pleasant, MI, United States; ^2^Department of Psychology, Central Michigan University, Mount Pleasant, MI, United States; ^3^Program in Neuroscience, Central Michigan University, Mount Pleasant, MI, United States; ^4^Department of Neuroscience, Karolinska Institute, Stockholm, Sweden; ^5^Field Neurosciences Institute, Ascension St. Mary's, Saginaw, MI, United States

**Keywords:** neurological disorders, hiPSCs, neural organoids, vascularization, blood-brain barrier

## Abstract

Modeling neurological disorders is challenging because they often have both endogenous and exogenous causes. Brain organoids consist of three-dimensional (3D) self-organizing brain tissue which increasingly is being used to model various aspects of brain development and disorders, such as the generation of neurons, neuronal migration, and functional networks. These organoids have been recognized as important *in vitro* tools to model developmental features of the brain, including neurological disorders, which can provide insights into the molecular mechanisms involved in those disorders. In this review, we describe recent advances in the generation of two-dimensional (2D), 3D, and blood-brain barrier models that were derived from induced pluripotent stem cells (iPSCs) and we discuss their advantages and limitations in modeling diseases, as well as explore the development of a vascularized and functional 3D model of brain processes. This review also examines the applications of brain organoids for modeling major neurodegenerative diseases and neurodevelopmental disorders.

## Introduction

Neurons and glial cells (astrocytes, oligodendrocytes, and microglia) are major cellular types of the central nervous system, which are essential for normal brain function and are implicated in most neurological disorders. During brain development, neurons, astrocytes, and oligodendrocytes are derived from neuroepithelial cells, called neural stem cells (NSCs). Both neurons and glia are found to be affected in various neurodegenerative disorders, including Huntington's disease (HD), Alzheimer's disease (AD), and Parkinson's disease (PD) (Phatnani and Maniatis, 2015; Tao and Zhang, 2016; Blanco-Suárez et al., 2017; Fu et al., 2018). Although more abundant than neurons, astrocytes and oligodendrocytes develop from NSCs after neurogenesis. While microglia are present throughout the CNS and play important roles in the development and functions of CNS, they are not generated from NSCs (Schwartz, 1997). Microglia play an important role in normal brain function. They are involved in a wide variety of neurological and psychiatric disorders, such as multiple sclerosis (MS), amyotrophic lateral sclerosis (ALS), spinocerebellar ataxia, PD, HD, AD, brain ischemia, autism spectrum disorder, obsessive-compulsive disorder, and schizophrenia (Schwartz, 1997; Menassa and Gomez-Nicola, 2018; Zhao et al., 2018).

While the etiology and pathophysiology of many neurological disorders are largely unknown, it is generally acknowledged that these conditions are caused or affected by interactions of genetic and environmental factors (Kwon et al., 2016; Berson et al., 2018; De Boni and Wüllner, 2019; Li et al., 2019). While the human brain is the ideal model to study such disorders, there are obvious ethical and technical limitations preventing this, not the least of which include unavailability of healthy and diseased brain tissue, as well as the difficulties with *in vitro* culture or genetic manipulation of adult brain tissue (Lee et al., 2017). Although animal models are commonly used to mimic human diseases, the high failure rate of translating most animal-based research into successful treatments in the clinic underscores their inadequacy to accurately model all critical features that characterize most human neurological disorders (Wang, 2018). For example, microcephaly, a neurodevelopmental disorder which is characterized by reduced brain size, has been modeled in transgenic mice, which contain microcephaly-related gene mutations, but does not adequately simulate this disorder, even in terms of brain size (Barrera et al., 2010; Pulvers et al., 2010; Lancaster et al., 2013). Likewise, many pharmacological interventions that proved successful in animal models of human neurological disorders do not translate into effective treatments in clinical trials (Burke, 2007; Pulvers et al., 2010; Takao and Miyakawa, 2015). As a further example, none of the ~25 transgenic rodent models of HD can reproduce all neurodegenerative features and recapitulate the progression of this disease as presented clinically in HD patients (Pouladi et al., 2013; Bhalerao et al., 2020).

Fortunately, newly developed, human-derived *in vitro* models provide promising tools to overcome several of these limitations. For example, neurons and other cells, along with their transcriptional profiles, which are generated from human-derived induced pluripotent stem cells (hiPSCs) can be used to simulate a fetal brain. Human embryonic stem cells (hESCs) and hiPSCs are similar in their cell morphology, proliferation, and differentiation capacity, although some DNA methylation profiles are changed in iPSCs because of the reprogramming process (Deng, 2010; Kim et al., 2010; Liang and Zhang, 2013). Moreover, hiPSCs are scalable, reproducible models, which are capable of recapitulating complex neurodevelopmental events during early embryogenesis and disease pathogenesis. Thus, the generation of patient-specific hiPSCs through cellular reprogramming can closely recapitulate disease manifestations of the clinical phenotypes observed in patients (Wu et al., 2019). The main advantages of using hiPSCs, compared to hESCs, are that they are patient-specific, which eliminates the risk of immunological rejection when they are obtained from the patient who will receive the transplant (Ho et al., 2018).

Currently, organoids are most often used to model the development and pathological alterations in various human organs, including the brain (Völkner et al., 2016; Czerniecki et al., 2018; Kim et al., 2019). Neural organoids that have been derived from differentiating iPSCs have been developed by using different methods (Lancaster et al., 2013; Jo et al., 2016; Qian et al., 2018). The 3D brain organoid consists of various cell types that can recapitulate cortical neuronal layers, cellular compartmentalization, and brain-like functions. In addition, such organoids can recapitulate the development of embryonic tissue more accurately than that of the 2D culture of hiPSCs. Thus, 3D organoids have more promise for the investigation of human brain development and complex human diseases, including neurodevelopmental and neurodegenerative disorders. In this review, we first present different 2D culture methods that are used to generate various neuronal and non-neuronal cells derived from hiPSCs. Next, we discuss the organoid technologies that use iPSCs as *in vitro* models of neurological disorders. Finally, we examine recent advances in 3D neural organoid technologies, as well as their applications and limitations as model systems.

## Recapitulation of NSCs and Neuronal Subtypes From iPSCs

Neurons are generated first during brain development, although the exact number of neural subtypes in the immature brain is unknown (Phatnani and Maniatis, 2015). Most neurological diseases are associated with damage to specific neural subtypes, such as midbrain dopamine neurons in PD (Luk et al., 2012), medium spiny GABA neurons in the striatum in HD (Reinius et al., 2015), and motor neurons in ALS (Kanning et al., 2010). Therefore, modeling neurological diseases using hiPSCs has enhanced our understanding of how those neural subtypes alter their function in each of these disease processes. Following multiple protocols in 2D cultures, NSCs, and then neurons and glial cells, are generated from hiPSCs (Chambers et al., 2009; Zheng et al., 2018b; Soubannier et al., 2020) as can be seen in Tables 1, 2. When hiPSCs are grown without medium components that promote self-renewal, embryoid bodies (EBs) are formed, and when fibroblast growth factor (FGF) or bone morphogenetic protein (BMP) inhibitors are absent, most hiPSCs form neuroepithelia (NE) or neural stem cells (NSCs) (Reubinoff et al., 2001). However, a single BMP or dual-SMAD inhibition method can be more useful than the EB method, due to culture variability. While the single BMP or dual-SMAD inhibition methods are equally effective in inducing neural differentiation, the dual-SMAD inhibition method with hiPSCs is more efficient in terms of forming neural rosettes (Zhang et al., 2018).

**Table 1:** Differentiation of NSCs and neuronal subtypes from human iPSCs.

**Generation of cell type**	**Origin**	**Factors inducing hiPSCs into NSCs**	**Factors inducing NSCs into neuronal subtypes**	**Characterization**	**References**
NSC	hiPSC	Dual-SMAD inhibition together with bFGF, N2, B27, ascorbic acid, and PMA		NSC expresses Nestin and Sox2, expression of Pax6, OCT4, and SSEA4 in hiPSC, and NSC forms neural rosettes and differentiates into neural lineages	Chambers et al., 2009; Wattanapanitch et al., 2014; Palm et al., 2015
**Neurons**					
GABAergic neurons	hiPSC-derived NSCs		BMPRIA, Dkk1, and PM	Neurons express GABA and VGAT	Nicholas et al., 2013
Cortical neurons			FGF-2 or PD0325901, SU5402, and DAPT	Expression of FOXP2 and SATB2 in cortical neurons	Shi et al., 2012; Qi et al., 2017
Motor neurons			SAG, FGF-2, and RA	ChAT positive neurons	Maury et al., 2015
DA neurons			FGF-8, RA, and N2 together with BDNF, GDNF, and dCAMP	TH and FoxA2 positive neurons	Hartfield et al., 2014
Serotonin neurons			SHH and FGF-4	Neurons express TPH-2	Liu et al., 2018a,b

*NSC, neural stem cells; DA, dopaminergic; bFGF, basicfibroblast growth factor; FGF-8, fibroblast growth factor-8; RA, retinoic acid; N2 supplement; BDNF, brain-derived neurotrophic factor; GDNF, glial-derived neurotrophic factor; dCAMP, dibutyryl cyclic adenosine monophosphate; SHH, sonic hedgehog; FGF-4,fibroblast growth factor-4; BMPRIA, bone morphogenetic protein receptor; Dkk1,Dickkopf-related protein 1; PM, purmorphamine; TPH-2, tryptophan hydroxylase-2; TH, tyrosine hydroxylase*.

**Table 2:** Differentiation of glial subtypes from human hiPSC-derived NSCs.

**Generation of cell type**	**Origin**	**Factors inducing NSCs into astrocytes**	**Factors inducing NSCs into OPCs**	**Factors inducing OPCs into Oligos**	**Characterization**	**References**
Astrocyte	hiPSC- derived NSCs	Astrocytic differentiating medium with 1-2 % FBS			Expression of GFAP, S100β, and AQ4.	Palm et al., 2015; Tcw et al., 2017; Soubannier et al., 2020
Oligodendrocyte	hiPSC- derived NSCs		RA and SAG or Sox10, olig2 and NKX6.2	PDGF-AA, NT3, and IGF-1	Expression of SOX10, PDGFRα, OLIG2, and NKX2.2	Douvaras et al., 2014; Ehrlich et al., 2017

*OPC, oligodendrocyte progenitor cell; Oligo, oligodendrocyte; FBS, fetal bovine serum;PDGF-AA, platelet-derived growth factor-AA; IGF-1, insulin growth factor-1; NT3, neurotrophin-3*.

The dual-SMAD inhibition method is a procedure that inhibits SMAD-dependent transforming growth factor-beta (TGFβ) and BMP signaling pathways with SB431542 and noggin. This efficiently converts hiPSCs to NSCs, which are characterized by specific markers (Table 1). In this method, human iPSCs are cultured with EB medium for 5 days and the medium is replaced with a neural induction medium for the next 4–14 days (Zhang et al., 2018). To produce mature neurons, EB-derived rosettes can then be re-plated on poly-ornithine/laminin-coated plates and cultured using a neural differentiation medium for several weeks (Zhang et al., 2018). Specific neuronal subtypes from iPSCs have been generated by using a neural differentiation medium that contains various inducing factors, as can be seen in Table 1. Because the ability to induce several types of neurons in culture is critically important for receiving, processing, and transmitting the information through their self-created networks and because neurodegenerative diseases may affect many of these neuronal connections, the production of different subtypes of neurons from patient-derived iPSCs has been crucial to characterize disease phenotypes (Wu et al., 2019; Tran et al., 2020).

### Recapitulation of Glial Subtypes From hiPSCs

Glial cells include oligodendrocytes, astrocytes, and microglia, which constitute almost half of the brain size (Azevedo and Feldman, 2010). Glia play important roles in many aspects of CNS, including brain development, homeostasis, and protection of neurons from brain injury and diseases (Reemst et al., 2016). Several neurological disorders, such as MS and Guillain–Barre syndrome, have been linked to glial cell dysfunction (Notturno et al., 2008). Here we describe current protocols for deriving various glial cells from hiPSCs and indicate how these glial cells can be used for future disease-modeling applications.

### Astrocytes

Generating astrocytes in a 2D culture of hiPSCs takes longer than 3 months because the process involves the induction of cell-fate decisions. The EB or dual-SMAD inhibition method is usually used to differentiate hiPSCs into NSCs and then for differentiating neuronal and glial subtypes (Chambers et al., 2009). Currently, there is no effective method that can circumvent neurogenesis and/or promote direct generation of glia. Therefore, NSCs need to be expanded until the onset of glial development, which is characterized by expression of NF1A, S100β, CD44, and downregulation of PAX6 expression, which usually occurs during the third month of hiPSC differentiation (Krencik and Zhang, 2011). Thereafter, the glial progenitors are differentiated into astrocytes under specific differentiating conditions, such as BMPs and CNTF (ciliary neurotrophic factor), which induce astrocyte differentiation by activating the STAT3 pathway (Rajan and McKay, 1998). While glial progenitors are generated by the end of the 3rd month, another 3 months are required to generate functional astrocytes (Krencik and Zhang, 2011).

Recently, some researchers recommend using protocols that differentiate astrocytes from hiPSCs with the expression of GFAP and S100β more quickly (Palm et al., 2015; Ben-Reuven and Reiner, 2020; Soubannier et al., 2020) (Table 2). Like neurons, astrocytes are also classified into many subtypes because of their location, morphology, and molecular /physiological functions. For example, fibrous astrocytes and protoplasmic astrocytes are found in white matter and gray matter, respectively (Wang, 2018). However, the current protocols need further improvement to produce regional-specific astrocytes from hiPSCs, which can be obtained by the modulation of RA, BMPs, and sonic hedgehog (SHH) in differentiating astrocytes, which, in turn, were derived from NSCs. Astrocyte dysfunction may be crucial in the malfunction of neurons (Phatnani and Maniatis, 2015; Garwood et al., 2017) and display disease-related phenotypes in AD and HD patients (Khakh et al., 2017). Thus, a further improved protocol may lead to the differentiation of mature and regionally-specific astrocytes, which would be a critical improvement for future applications in disease-modeling.

### Oligodendrocytes

During development, oligodendrocytes start to appear from NSCs later than neurons and astrocytes and provide support through the formation of myelin sheath that wraps around and insulates the axons in the CNS. While the origin of oligodendrocytes includes the ventral neural tube, the dorsal neural tube, and the subventricular zone (SVZ) during development (Wang et al., 2012), most studies have focused on the area, such as the ventral part of the telencephalon and spinal cord, where the oligodendrocyte progenitor cells (OPCs) are differentiated from NE in response to SHH-induced expression of the Olig1/2 gene (Tao and Zhang, 2016). To produce oligodendrocytes, iPSC-derived NSCs are moved to a glial-induction media, containing platelet-derived growth factor-AA (PDGF-AA), insulin growth factor-1 (IGF-1), and Neurotrophin-3 (NT3) (Wang et al., 2012). Gliospheres can be derived from these NSCs in presence of these factors, which can be re-plated in smaller sizes to differentiate into oligodendrocytes. To date, these essential steps have been followed to differentiate oligodendrocytes from hiPSCs (Ehrlich et al., 2017). Similar to what is observed in brain development, neural specification is the first step of oligodendrocyte differentiation from hiPSCs, which is the same as that for differentiation of neurons and astrocytes (Table 2). The duration of differentiation of iPSCs into oligodendrocytes varies from 110 to 150 days.

Recently, Ehrlich et al. (2017) demonstrated a rapid and efficient protocol for generating oligodendrocytes from hiPSC-derived NSCs by inducing three transcription factors (SOX10, OLIG2, and NKX6.2). This method yields up to 70% O4+ expressing oligodendrocytes within 28 days of differentiation and 30% of these cells differentiate into mature myelin basic protein positive (MBP+) cells within 35 days. However, induction of specific transcription factors may interrupt the production of disease phenotypes of patient-derived iPSC models. Therefore, the simplest solution would be finding a mitogen that is neutral to the fate of the differentiation process undergone by the progenitor cell, which would be a crucial feature for a stable organoid model.

### Microglia

Microglia are known as macrophages of the CNS, which account for 5–10% of total neural cells in parenchyma (Zhao et al., 2018). They are uniformly distributed throughout the brain. While the identity of microglial progenitors remains controversial, it is hypothesized that microglial differentiation has occurred in the CNS from embryonic and perinatal hematopoietic cells. Immune defense and maintenance of homeostasis are two key functions of microglia in the CNS. They are required for inflammatory responses of the CNS, and they are dysregulated in several different diseases, including AD and PD (Zheng et al., 2018b; Song et al., 2019a,b). In recent years, different protocols have been proposed to generate microglial cells from iPSCs (McQuade et al., 2018; Menassa and Gomez-Nicola, 2018). Colony-stimulating-factor-1 (CSF1) receptor ligands are available in all these protocols, which are required for the proliferation, differentiation, and survival of normal macrophages. However, batch-to-batch variability and cellular heterogeneity are the limiting factors for the use of these methods (Quadrato et al., 2017; Pollen et al., 2019).

To bypass the formation of EBs, Abud et al. (2017) proposed a different protocol for the differentiation of hematopoietic progenitors directly from iPSCs, which were further differentiated into functional microglial-like cells. Recently, the earlier methods have been improved by adding IL-34 and granulocyte-macrophage colony-stimulating factor (GM-CSF), instead of CSF1 (Song et al., 2019b). This method provides higher cell proliferation (higher BrdU+ cells) and reduced ROS expression and produces a more accurate simulation of the tissue-specific microenvironment. The co-culture of the microglia-like cells (MG) with different neural organoids produces different migration patterns and functional activities, including variation in response to exogenous factors. In addition, transcriptome analysis revealed that microglia-related genes are expressed differently in MG when they are co-cultured with neural organoids (Song et al., 2019b). These findings indicate that generating microglial from iPSCs is a significant advance in iPSC technology, whereby non-neuronal cells can be readily derived from iPSCs, providing an avenue for more efficient designs of organoid models in the future.

## The Blood-Brain Barrier (BBB), Brain Microvascular Endothelial Cells (BMECs), and Pericyte Production of iPSCs

The precise organized activity among different cell types within the neurovascular unit (NVU) is required to maintain CNS functions (Zhao et al., 2015). The interaction of NVU components, such as pericytes, endothelial cells, smooth muscle cells, astrocytes, oligodendroglia, microglia, and neurons is critical for the energy demands of the brain. The blood-brain barrier (BBB) is an important part of NVU, which consists of unique microvascular endothelial cells (BMECs), neurons, astrocytes, microglial cells, and pericytes. These multiple cell types are interconnected by tight and adherent junctions and express specific molecular transporters (Benson and Joseph, 1961). Furthermore, these dynamic cellular complexes are essential for brain homeostasis and regulate active interaction between the bloodstream and CNS. The transport of essential molecules and nutrients into brain is regulated by the BBB, which is required to maintain an optimal CNS function (Fernández-López et al., 2012) by responding to many physiological and pathological cues (Erickson et al., 2020). Thus, the complex vascular network of the BBB protects the CNS from not only systemic fluctuations but also various harmful substances, including pathogens and toxins (Bhalerao et al., 2020).

BMECs form the innermost layer of the vasculature, which is characterized as a physiological barrier, and active metabolic system. Therefore, these unique cell types can regulate tissue microenvironment by synthesizing various materials. Structural and functional alterations of the BMECs have been reported in several neurological disorders, such as stroke, traumatic brain injury, and neurodegenerative diseases (Liu et al., 2018a,b; Jarazo et al., 2019). BMECs malfunction when they are removed from the brain microenvironment and cultured for extended periods of time. Fortunately, new iPSC technologies are opening up unique opportunities to generate BMECs that can be used in the modeling of various diseases with different components of the BBB.

The first iPSC-derived BMEC population was detected by co-differentiating neural-like and endothelial cells (Lippmann et al., 2012). Thereafter, several protocols have been published with modification of cell culture conditions, with more efficient differentiation processes, and with the integration of the specific disease pathology (Canfield et al., 2017; Erickson et al., 2020). Canfield et al. (2017) first demonstrated that iPSCs can generate functional BMECs, in conjunction with neurons and astrocytes. When BMECs are co-cultured with neurons and astrocytes, the barrier tightens, and tight junction localization is significantly improved. Furthermore, the iPSC-derived BBB can be used as a model by improving functional properties, such as improved transporter activity, barrier tightening, tight-junction localization, and paracellular permeability (Appelt-Menzel et al., 2017; Hollmann et al., 2017). However, the generation of BBB components from hiPSC and precisely coordinated activity among these dynamic cellular complexes in real-time is needed for the reproducibility and application of iPSC-derived multicellular BBB models.

Pericytes are part of the NVU, with stem cell-like properties, and consist of multi-functional cells located inside capillaries throughout the body, including the brain. They are critical for the regulation of cerebral blood flow, BBB, neuroinflammation, vascular development, and angiogenesis. Pericyte dysfunction may induce vascular diseases, such as stroke and neurodegenerative diseases. These cells may also cause transcytosis across the interior of BMECs (Armulik et al., 2010). As multi-faceted mural cells, pericytes are found in smaller vascular structures, including capillaries, and are characterized by specific markers, such as platelet-derived growth factor receptor-beta, smooth muscle protein 22 alpha, and calponin-1 (Armulik et al., 2011). During development, neural crest stem cells give rise to CNS mural cells, while peripheral mural cells are generated from a mesodermal lineage (Etchevers et al., 2001). CNS mural cells can also be produced from iPSCs, but their functionality as part of the BBB is still unknown.

A recent study demonstrated an updated protocol to produce brain pericyte–like attributes from hiPSC-derived neural crest stem cells (NCSCs) (Stebbins et al., 2019), which can integrate with endothelial cells to form vascular networks. Moreover, these cells have been characterized as scalable and reproducible, with BBB properties. Thus, the generation of pericytes from patient-derived iPSCs delivers a unique tool for the investigation of CNS disorders, including stroke, epilepsy, demyelinating disease, and AD (Stebbins et al., 2019).

The generation of patient-specific iPSCs can recapitulate disease phenotypes that are reported in patients. The iPSC-based 2D protocols have been used to investigate the pathophysiology of specific cell types that are involved in neurological diseases. However, these methods are still unable to provide the complex organization that mimics such complicated processes as embryonic development, cellular networking, and disease development (Dang et al., 2016). Moreover, the diversity of iPSC-derived cells and various types of neuronal and glial precursors are too immature to provide the metabolic, structural integrity, and neuronal activity that is observed in the mature brain. The genomic and epigenomic signatures are also more vulnerable to time-dependent alterations in 2D culture systems.

In this context, hiPSC-derived 3D brain organoids are more attractive tools for *in vitro* disease modeling because they can undergo multi-lineage differentiation and self-organization to form heterogeneous cell populations and tissue-like architecture (Lee et al., 2017). This 3D environment is more conducive to the differentiation of specific cell types that can mimic the early stages of human CNS maturation. Gene regulatory mechanisms observed during neurogenesis in the primary cortex are more accurately recapitulated in organoid models, which further support their utility. In addition, 3D brain organoids are appropriate tools for drug screening and tissue replacement therapy, especially in the context of neurodevelopmental and neurodegenerative disorders.

## Current Protocols for the Generation of 3D Organoids and Self-Patterning of hiPSCs

During brain development, the patterning of anterior-posterior (A-P) and dorsal-ventral (D-V) axes are regulated by specific signaling molecules. For example, FGFs, retinoic acid (RA), and WNT signaling regulate the A-P patterning, whereas Wntss, BMPs, and SHH influence D-V patterning. The most common method of simulating brain development processes involves the generation of neurons from hiPSCs by producing NE or NSCs as a preliminary step toward differentiating neural subtypes as a function of activating specific types of growth factors or signaling pathways. Either the single BMP or the dual-SMAD inhibition approach can produce NE from hiPSCs, allowing certain region-specific markers, such as paired box 6 (PAX6) and orthodenticle homeobox 2 (OTX2), to be expressed in an anterior part of the brain (Zhang et al., 2018). Later, this NE gives rise to a specific brain region within 2 weeks in presence of morphogens. For example, CHIR99021 (CHIR), which activates the Wnt pathway, can establish an incrementally increased concentration gradient that can differentiate the NE into different brain regions (forebrain, midbrain, and hindbrain) as a function of its increased concentration along the A-P axis (Kirkeby et al., 2012). A specific concentration of CHIR can generate specific neuronal populations, such as midbrain DA neurons and hindbrain serotonin neuronal populations. Following these principles, the combination of Wnt, RA, and FGFs on NE can also generate spinal cord cells (Maury et al., 2015).

The SHH, the WNT canonical pathway, and the BMP pathway regulate D-V patterning. These signaling molecules are secreted from the notochord and/or the floor plate. In absence of SHH signaling, the anterior NE give rise to the cerebral cortical identity (Le Dréau and Martí, 2012). The NE give rise to the lateral ganglionic eminence (LGE) when the expression of SHH is low. The specific markers, GS Homeobox 2 (GSX2), B-cell CLL/lymphoma 11B (CTIP2), and Meis homeobox 2 (MEIS2) are expressed in this region. Primarily, the medium spiny GABA neurons are produced from these progenitors (Arber et al., 2015). In the presence of a high concentration of SHH, the NE become progenitors for the medial ganglionic eminence (MGE), which is identified by the expression of NK2 homeobox 1 (NKX2.1). These progenitors differentiate into GABAergic interneurons and forebrain cholinergic neurons (Liu et al., 2013; Kim et al., 2014). Thus, the production of different cell types that forward the D-V axis is regulated by the level of SHH and /or a balance between SHH and Wnt pathways.

As an example of this D-V patterning, radial glia progenitor cells first generate neurons in cortical layer VI and I during the development of the cerebral cortex. Then these progenitors gradually differentiate into neurons in layers V, IV, III, and II (Gaspard et al., 2008). Based on morphogen availability along D-V axes during brain development, the most common approach for simulating neuronal induction from hiPSC involves the dual-SMAD inhibition factors, noggin and SB431542, and the Wnt pathway activator, CHIR. Later, SHH along with either RA or FGF8, is added to the culture medium to produce cerebral or midbrain patterned organoids, respectively. Brain-derived neurotrophic factor (BDNF), and glial cell-derived neurotrophic factor (GDNF) are important for the maturation, function, and survival of different types of neurons, including cholinergic, dopaminergic, serotonergic, and gamma-aminobutyric acid (GABA)-ergic neurons. Along with these specific factors, other complementary molecules are included in the medium to facilitate organoid development, including rho-associated protein kinase (ROCK) inhibitors for the survival of iPSCs, heparin for enhancing the activity of Wnt signaling, 2-mercaptoethanol for the reduction of oxidative stress and cell death, laminin and insulin for the tissue growth, and ascorbic acid and DB-cAMP for enhancing neuronal differentiation.

Based on the protocol developed by Lancaster et al. (2013), iPSCs can be differentiated into organoids between 1–2 months and maintained for up to a year, although the rate of growth is reduced gradually after 2 months due to necrosis inside the organoids (Lancaster et al., 2013). Later, Lancaster et al. found that agitation of the organoids using a spinning bioreactor decreased necrosis and increased the rate of organoid growth. Such improvement of their growth could be due to the availability of sufficient oxygen and nutrients during the differentiation process (Lancaster and Knoblich, 2014). Although comparing neurodevelopmental sequelae of organoids with those that occur in humans is somewhat speculative, given our current knowledge, it does appear that a 2-month-old organoid brain parallels the neuronal development occurring during the first trimester in humans (Lancaster et al., 2013). Cerebral organoids at 2 months contain NSCs, astrocytes, neurons, and synaptic structures, suggesting increased cellular diversity and neuronal organization.

The field of neural organoids has been advanced and made more sophisticated by the modification of current protocols and applying advanced technologies in recent years (Kelava and Lancaster, 2016). For example, the methylome and transcriptome profiles of cerebral organoids possess significant similarities to human fetal brains (Luo et al., 2016), although further studies are required to ensure stable reproducibility (Luo et al., 2016). More recently, the development and characterization of vascularized organoids have been one of the most significant advances in this field (Mansour et al., 2018).

While chemical signals can be used to manipulate most of the changes in the self-organization of organoids, there still remain several shortcomings to overcome. It is difficult to predict how these current methods regulate spatiotemporal patterning and differentiation in neural organoids, given that critical morphogens can be secreted endogenously, including BMPs and Wnts, as well as SHH and RA. Recently, Lancaster et al. (2017) showed that poly (lactide-co-glycolide) copolymer (PLGA) fiber microfilaments can be used to produce enlarged EBs. Moreover, organoids produced in these conditions not only lengthen the NE production, but also improve the formation of neuroectoderm, cortical layers, and even increase reproducibility (Lancaster et al., 2017). One new development involves fusing organoids. For example, organoids with dorsal identity are fused with those with a ventral identity to form a D-V axis (Xiang et al., 2017). However, a D-V axis can also be formed in a single organoid and display a self-organized cortical plate as Lancaster et al. (2017) demonstrated earlier.

While current 3D organoid protocols have taken precedence over 2D methods in the context of cellular diversity, neuronal connection, and tissue-like functions, these models are still unable to respond appropriately to infection, toxic substances, and age-associated inflammation, due to their lack of vascularization and immune cells, such as endothelial cells, monocytes, macrophages, or leukocytes (Ho et al., 2018). The currently used organoids do not recapitulate the later stages of the human embryonic brain (Monzel et al., 2017; Pham et al., 2018) and to do this would require further improvement in culture methods that are more physiologically and biologically relevant in order to accurately model the human brain. The lack of cellular diversity, the establishment of circuits, cell viability, and maturation are the major concerns for current organoid development.

Crucially, the formation of a vascular system, which is essential for oxygen penetration, nutrient supply, and NSC migration and differentiation is needed. The deficiency of vascular dissemination can induce hypoxia in organoid cultures and may enhance necrosis in the center of the organoid, which can hinder the neurogenesis, their migration routes, and networking. In addition, current organoids are limited in their ability to exhibit cell–tissue interactions, patterns of neuronal networking, and integration of the brain immune system with organoids. Clearly, a more extensive method for establishing better vascularized neural organoids to model human neurodevelopment and neurological diseases is needed.

### Generation of Patient-Derived Organoids With Vasculature and Microglia Components

A vascularized organoid could recapitulate the *in vivo* vascularization process that occurs during brain development and may reveal new features that have not yet been observed. A critical component for recapitulating vascularized organoids is the BBB, which, as indicated above, is a highly selective border consisting of BMECs, basement membrane, along with pericytes, astrocytes, and microglia. Brain homeostasis is regulated by BBB, which is essential for neuronal activities. The murine CNS starts to form vascularization at embryonic day 7.5–9.5, whereas human brain vascular components are detected within a gestational week 7 (Cakir et al., 2020). ECs play a critical role for neuronal maturation of cortex (Cakir et al., 2020) as it provides a protective layer of tightly joined cells that prevent diffusing toxins and infectious agents into the surrounding brain tissue. Disruption of the BBB has been associated with numerous neurological disorders, including HD, in which the breakdown of the BBB is thought to accelerate the progression of the neuropathological processes in the R6/2 mouse model of HD (Di Pardo et al., 2017), and post-mortem tissue from HD patients (Di Pardo et al., 2017; Bhalerao et al., 2020).

**Figure 1:**
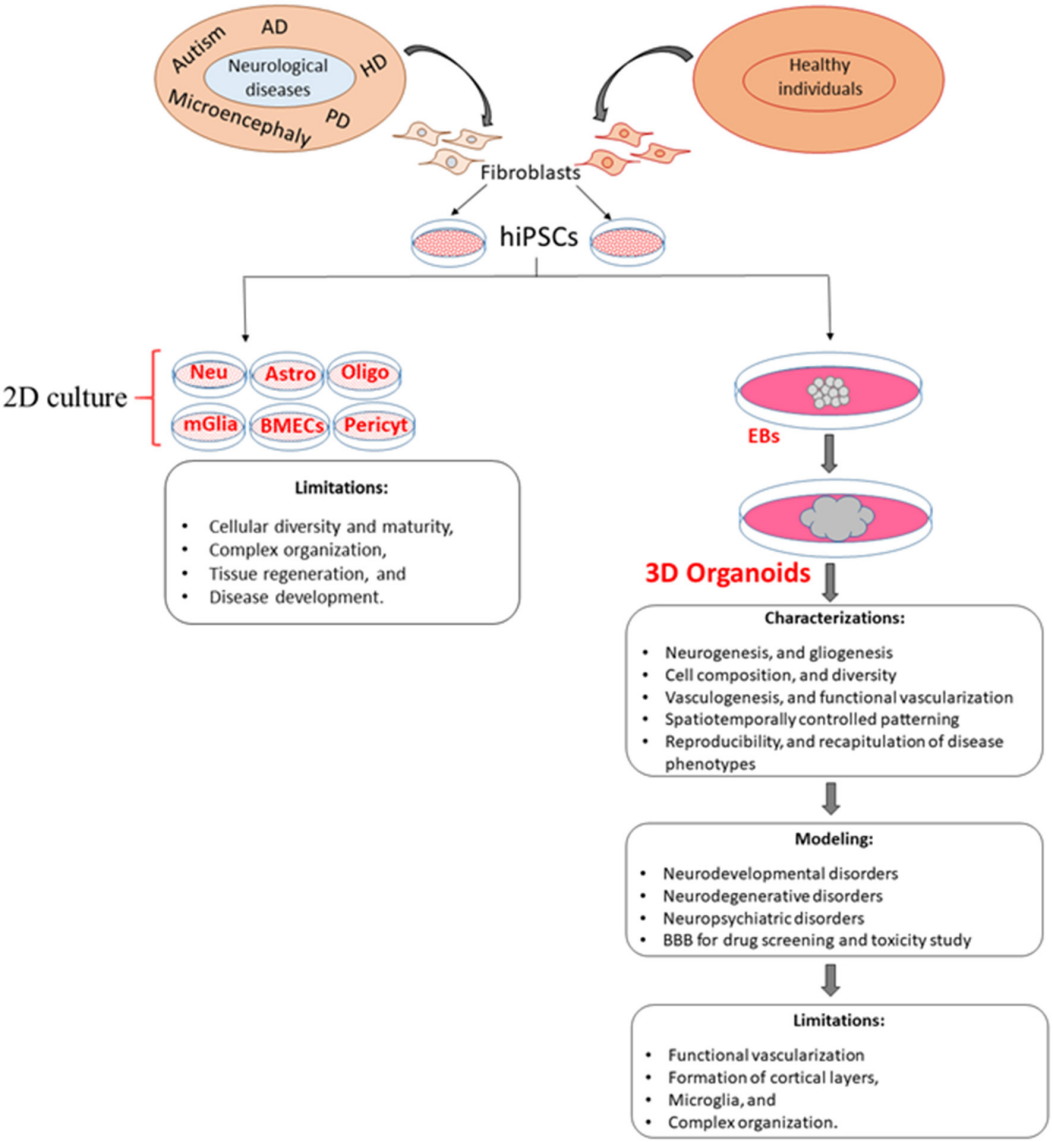
Comparison of 2D and 3D cell culture models and their limitations. Although 3D models overcome some of the limitations of 2D models, the generation of new 3D models is needed to increase vascularization, cellular diversity, viability, and reproducibility as well as a more accurate recapitulation of disease phenotypes. EBs, embryoid bodies; Neu, Neuron; Astro, astrocytes; Oligo, oligodendrocyte; mGlia, microglia; BMECs, brain microvascular endothelial cells; Pericyt, pericyte.

The recruitment of vascular networks, immune cells, and the BBB within organoids would make them a more physiologically relevant model of the human brain. Therefore, the generation of vascularized organoids has been recognized as a more relevant model to human diseases, which would allow for diffusing oxygen and nutrients throughout the mass that would support differentiation of multiple cell types into complex organization-like structures (Figure 1; Table 3). From a therapeutic perspective, a vascularized organoid could mimic normal physiological and pharmacological events that occur in the body, such as drug uptake, circulation, and metabolism. Although transplantation of human-derived nonvascular brain organoids into the rodent brain can produce integration, viability, long-term survival, increased angiogenesis, functional neuronal activity, and synaptic connectivity of the grafted organoid (Daviaud et al., 2018; Mansour et al., 2018), these beneficial effects are likely to be enhanced with transplants of vascularized organoids. In addition, a vascularized organoid developed from patient-specific hiPSCs might be grafted more successfully into patients, as it may integrate more readily with the vascular network of the patient (Ho et al., 2018).

**Table 3:** Current protocols for hiPSC-derived 3D vascularized organoids.

**Cell types**	**Organoid types**	**Characterization**	**Recapitulation of disease phenotypes**	**Limitations**	**References**
HiPSCs with HUVECs-derived ECs	Cortical	Lack of connection between human brain organoid capillaries with the rodent host brain	Not addressed	Limited regulation in Vasculature, and formation of microglia	Pham et al., 2018
HiPSCs with HUVECs-derived ECs	Cortical	Lack of ECs cluster and the functional BBB	Not addressed	Limited regulation in Vasculature, and formation of microglia	Shi et al., 2020
HESCs with the induction of ETS variant 2 (hETV2)	Cortical	Functional BBB, and maturation of neurons	Not addressed	Lack of functional blood vessels, the six distinct cortical layers, and the limited formation of microglia	Cakir et al., 2019

*HiPSCs, human induced pluripotent stem cells; HUVECs, human umbilical vein endothelial cell; HESCs, human embryonic stem cells*.

ECs can be generated from hiPSCs, which support the feasibility of producing and integrating a vasculature using current organoid models (Pham et al., 2018). Briefly, to produce these vascularized organoids, iPSCs were first cultured and differentiated to ECs using the Stem Diff APEL medium, supplemented with morphogenetic protein 4 (BMP4), FGF-2, and vascular endothelial growth factor (VEGF). Thereafter, ECs derived from iPSCs were co-cultured with the cerebral organoid for 3–5 weeks before transplantation, which resulted in robust vascularization of organoids within 5 weeks. However, the limitation of the transplanted vascularized organoids is that capillaries were found in the center of rosettes, as well as in between rosettes, although the presence of rodent blood in these capillaries was not verified, as the brains were perfused with saline and formalin (Pham et al., 2018). Moreover, proof of any connectivity between the human brain organoid capillaries and the rodent host brain is lacking (Pham et al., 2018), which is a hurdle in the combination of *in vitro* and *in vivo* models, as disease modeling should be maintained under physiological conditions. Human umbilical vein endothelial cells (HUVECs) are derived from the endothelium of veins of the umbilical cord, which form a tube, and have been widely used to characterize angiogenesis and other biological processes (Shi et al., 2020). More recently, another study reported that co-cultures of hESCs or hiPSCs with HUVECs generate human brain microvascular endothelial cells (HBMECs), which were characterized by P-glycoprotein (P-gp), and are the main ECs in the human brain, playing important roles in the BBB formation (Shi et al., 2020). However, evidence of ECs clustering and forming a functional BBB in the organoids is lacking (Shi et al., 2020).

ETS variant 2 (hETV2) is a transcription factor that is essential for the development of vascular endothelial cells. Based on this idea, Cakir et al. (2020), demonstrated a robust vascularization in cortical organoids, *in vitro*, with the induction of ETS variant 2 (hETV2). This vascularized organoid acquired several BBB characteristics, including an elevated expression of tight junctions, nutrient transporters, and trans-endothelial electrical resistance. Furthermore, the transcriptome profiles of neurons generated in non-vascularized and vascularized organoids were compared with human embryonic brains at gestational weeks 8–23. The transcriptome analysis of these neurons revealed that vascularized organoid-derived neurons resemble more mature neurons of a developing brain than that of non-vascularized organoids (Cakir et al., 2020). These results suggest that vascularization accelerates the maturation of neurons.

Recently, amyloid (Aβ1–42) oligos have been found to damage endothelial cells, tight junctions, and the BBB in AD (Wan et al., 2015). The biological function of BBB was further evaluated in this study by the deposition of Aβ peptide species (Aβ1–42-oligos or Aβ1–42-fibrils) in these vascularized organoids (Cakir et al., 2020). The results of this study indicated that Aβ1–42-fibrils do not damage tight junctions as effectively as Aβ1–42-oligos. Altogether, these results indicate that overexpression of hETV2 gene induces functional endothelial tight junctions and BBB-like characteristics. Nonetheless, several unresolved limitations remain, such as the lack of functional blood vessels, absence of the six distinct cortical layers, and the limited formation of microglia (Table 3), which play important roles in the formation of subventricular vascular plexus (SVP). Most notably, the absence of a vascular system can be detrimental to long-term organoid survival, as long-term culture of organoids demonstrates a continuous apoptotic cell death at the inner-most regions (Cakir et al., 2020). Moreover, neuron progenitor differentiation is impaired without a functional vasculature (Cakir et al., 2020).

Microglia and the neurovasculature display physical interactions, although they do not appear in the developing CNS at the same time. During brain development, microglia originate from the embryonic yolk sac, then migrate and colonize the neuroepithelium (Reemst et al., 2016). While several protocols have been developed to produce microglia-like cells or precursors derived from hiPSCs (Muffat et al., 2016; Abud et al., 2017; Song et al., 2019b), most microglia derivations lack a microenvironment that promotes region-specific microglia function. For example, forebrain microglia, but not cerebellar microglia, depend on IL-34 for maintenance. Microglial cells are found in the conjunction with the vasculature in the developing rodent brain and are sometimes referred to as “pericytic macrophages” (Thomas, 1999), which play critical roles in angiogenesis and the maintenance of the BBB integrity.

We mentioned earlier that different cell signaling molecules, such as Wnt, BMP, and SHH control the regional identity of the developing brain. While organoid technologies have been promising models to study *in vivo* brain development and disease phenotypes, the major limitation is to achieve truly *in vivo*-like functionality due to the lack of reasonable size and structural organization of tissues, such as the shape of the D-V, mediolateral, and A-P axes. Organoids are most often grown as floating in the culture medium, and morphogens are added to the medium uniformly for self-organizing and cell fate-patterning. However, compared to what occurs *in vivo*, organoids exposed to this conditional media lack concentration gradients of morphogens, which results in a stochastic organization of the developing organoids, rather than asymmetric brain development, such as the establishment of the D-V, mediolateral, and A-P axes.

While different protocols (Bagley et al., 2017; Wang et al., 2018) were established to develop the D-V and A-P axis in human brain organoids, morphogen-loaded beads were found to be more effective in establishing spatial identities in human brain organoids (Ben-Reuven and Reiner, 2020). In this method, organoids with morphogen-soaked beads were co-cultured to diffuse morphogens from the beads to the tissue, which create a concentration gradient of morphogen secretions in the developing organoids. For example, organoids embedded on Wnt and BMP4-soaked agarose beads may increase the local concentration of morphogens, which is decreased within the tissues. Making such gradient of morphogen availability allows for high and low concentration of morphogens to induce dorsal and ventral patterning of the developing brain organoids, respectively. However, providing vasculature and microglial integration would further improve the current status of organ-on-chip technology. In embryonic brain development, developing tissues can interpenetrate and interact with the complex vasculature system, which facilitates diffusion of oxygen, nutrient, and exchanging waste, as well as a structural template for growth. However, a perfect protocol for *in vitro* organoid development with the integration of vasculature has remained largely missing, which may be a crucial requirement for large-scale and more reproducible tissue organization, including D-V and A-P identity in the organoids. Meanwhile, microglial integration with organoids may enhance migration ability and immune response, which may facilitate maturation and neural circuit development. Different cutting-edge technologies, including 3D microelectrode arrays (MEAs) and optogenetics, have been used to characterize neural circuits. However, an advanced method for the development and characterization of vascularized organoids is needed.

Organoids that integrate microglial cells with ECs to develop vascularization should provide new insights into how microglia can interact with neurovascular systems to form a functional BBB, as well as how microglia-neuron and microglia-astrocyte communication function in both normal and pathological conditions (Zhao et al., 2018) (see Figure 1). Such newly developed systems could also facilitate replicating organoids of the same size and shape, as well as similar cellular composition, phenotypic and molecular characteristics (Lee et al., 2017), along with greater integration of vascular endothelial cells, immune cells, microglia, and BBB within organoids.

### Organoid on ChiP

Recently, organ-on-a-chip technology has captured the attention of researchers and inspired the development of a new complement to current organoid models. This technology is a promising tool for disease modeling, drug screening, and toxicity testing. Organ-on-chip systems have become more advanced because of using microfluidic technology. Therefore, different microfluidic organ-on-chip systems have been developed to model various organs, including the liver, kidney, intestine, lung, heart, and brain. Recently, Zhang et al. (2017) built a device that has physical, biochemical, and optical sensing capabilities to regulate the cellular microenvironment for recapitulating complex organization and functions. Subsequently, Wang et al. (2018) developed a perfusion-based organoid culture on a microfluidic chip where the EB is matured into self-organized organoids under a controlled microenvironment, such as diffusion of oxygen and nutrient to the growing cells and removing of waste from these cells. Such a microenvironment is essential for self-organized organoids to mimic the *in vivo* organ structure and function, which currently is lacking unconventional organoid models.

Recent advances of these devices provide more transparent *in situ* real-time imaging of the neurodevelopmental process and the brain organoid responses to exogenous factors, including nicotine, valproic acid, and cannabis (Wang et al., 2018; Ao et al., 2020; Cui et al., 2020), suggesting an alternative approach for characterizing 3D organoids in the human neurodevelopmental disorders. The new edition of these devices can integrate multiple organs for simulation, and pharmacokinetic assays for new drugs (Skardal et al., 2017). In addition, multi-organ chips are important systems for studying the efficacy and toxicity of new drugs, interaction among different organs, and developing drugs that are tailored to specific individuals (Skardal et al., 2017; Schimek et al., 2020).

While these microfluidic platforms can improve brain organoid uniformity by minimizing size variation, as well as hypoxia and cell death inside of the organ due to the availability of a controlled microenvironment as mentioned above, the system still cannot recapitulate fully the physiological and disease features that occur *in vivo*. Difficulties in standardizing and scaling up these types of organoids remain formidable challenges. In addition, a means fo producing microglia and a functional vasculature are lacking in those devices, which impede the development of mature and functional organoids, which are important for simulating the later stages of *in vivo* neurodevelopment and disease features.

### Characterization of 3D Organoids With Cutting-Edge Technologies

Characterization of many developmental and disease-specific features of these organoids has been challenging due to lack of proper technologies, while several cutting-edge technologies have been used to characterize the organoids such as MEA, patch-seq, and optogenetics. The potential application and limitations of these methods in 3D organoids are briefly discussed below.

**Microelectrode arrays (MEA)** Non-invasiveMEA recordings are often used to investigate the effects on neuronal impulse activity and to analyze the underlying ion currents in isolated individual neurons. The design of MEAs for 2D cultures of neurons is different from 3D cultures of neurons, while recordings of neuronal action potentials are common between these two arrays. The design of such 3D MEAs is challenging because the materials used need to be sufficiently pliable to avoid damaging the tissue. Polyimide is a good choice because of its favorable thermal and chemical tolerance and it can be integrated without causing 3D tissue damage. While *in vitro* culture of 3D organoids has been advanced, lack of appropriate 3D MEA methods for precisely monitoring and modulating neuronal activities in organoids remains a challenge. Recently, Soscia et al. (2020) designed 3D MEAs which can provide multi-channel recordings as well as record precise signals that are appropriate to the cellular morphology, neural network dynamics, and drug responses. Recent advances of 3D MEAs have demonstrated integrated capabilities to stimulate surrounding neurons to form a neural network in real-time in such 3D neural organoid models (Shin et al., 2021). However, further refinements of these 3D MEAs, with appropriate size and density of electrodes to accommodate 3D organoids is needed.

**Optogenetics** is based on genes coding for light-sensitive proteins (opsins) and the expression of these genes requires a specific color of light. For example, channelrhodopsin-2 (ChR2) expression is turned on by blue light and halorhodopsins are active in dark conditions. In this method, a combined (optical and genetic) tool is used to regulate individual neuronal activity. This technique has been successfully applied to embryonic stem cells (ESCs) to study motor deficits (Baker et al., 2016). Quadrato et al. (2017) performed single-cell sequencing and analyzed the data to demonstrate that light stimulation of photosensitive cells could regulate neuronal activity within organoids. This tool has also been used in grafted cerebral organoids (Mansour et al., 2018) and cortical spheroids (Yoon et al., 2019) to study host-graft functional connectivity, and express opsin by blue light. Thus, this technique could be a very useful tool to elucidate light-sensitive tissue organization and functional human neuronal circuits in the developing human brain. Finally, a combination of both the organoid system and optogenetic technology may allow neuroscientists to study morphological and functional diversity of neural circuits precisely in the healthy and diseased brain tissue.

**Patch-seq** can be considered as a different approach from high-throughput droplet-based single-cell RNA sequencing (scRNAseq), which has revolutionized neuroscience. In this technique, first functional properties of a specific neuron are measured by patch-clamp electrophysiology before studying the molecular basis of morphologic and functional diversity using transcriptional profiling of the cell. Currently, the technique has potential application in a variety of models, including hiPSC-derived neurons (Yoon et al., 2019). While Patch-seq has been used for *in vivo* recordings (Cadwell et al., 2016; Liu et al., 2020), the cell culture-based approaches make it easier for identifying live cells by high-resolution imaging and increases the capability of collecting RNA from the entire neuron. However, the significant challenges of this technique are that it is laborious and has a relatively low throughput compared to droplet-based scRNAseq. An advanced improvement of patch-clamp recordings and sequencing methodologies will certainly extend the application of Patch-seq methods in organoid technology.

### Recapitulation of Neurodegenerative and Neurodevelopmental Disorders Associated With the Genetic Signature in Patient-Derived Organoids

Organoids that have been derived from hiPSCs from patients with neurodegenerative disorders, such as HD, AD, and PD, or neurodevelopmental disorders, such as microencephaly and autism spectrum disorder, can be useful tools for understanding the pathophysiological mechanisms of these disorders.

**Huntington's disease** is a devastating neurodegenerative disease, which is characterized by motor dysfunction, cognitive impairment, and psychiatric symptoms (Walker, 2007; Zheng et al., 2018a). The major cause of HD is a mutation in exon 1 of the *HTT* gene, while other factors, such as environmental factors, may be involved in the progression of this destructive disorder. The mutation of the *HTT* gene results in an expansion of cytosine-adenine-guanine (CAG) trinucleotide repeats (TNR) (MacDonald et al., 1993). These CAG repeats form abnormal aggregates, which become toxic to both the neuronal population and their neural networks by interfering with the cellular machinery, including axonal transport, mitochondrial, and synaptic function. These toxic aggregates initially damage the medium spiny neurons (MSNs) of the striatum. To date, various 2D culture methods have been used to generate MSNs from hESCs and human iPSCs as MSNs are the major susceptible cell type in HD (Adil et al., 2018; Wu et al., 2018, 2019). Although 2D culture methods provide highly enriched MSNs from HD patient-derived iPSCs with higher than 50 CAG repeats for disease modeling, the recapitulation of HD-relevant phenotypes, including neuronal degeneration, often requires the addition of other cellular stressors. For example, mitochondrial dysfunction and oxidative stress occur when HD-iPSC-derived neurons are exposed to oxidative stress-inducing molecules. Recently, Conforti et al. (2018) produced a 3D cortical organoid using hiPSCs derived from HD patients, which was characterized by developmental defects in ventral-telencephalic and striatal formation during maturation. However, this model failed to show HTT protein aggregation and cell viability as the main pathogenesis of HD, suggesting that more work is needed to ensure that patient-derived organoids accurately recapitulate the disease phenotype (Table 4).

**Table 4:** Modeling neurodegenerative diseases in hiPSC-derived organoids.

**Cell types**	**Organoid types**	**Characterizations**	**Recaptitulation of disease phenotypes**	**Limitations**	**References**
HD-hiPSCs with more than 50 CAG repeats	Cortical	Abnormal neural cell positional identity, self-organizing, and maturation	Not addressed	Lack of Vasculature and microglia formation	Conforti et al., 2018
FAD-hiPSC with PSEN1 mutation FAD or DS-hiPSCs	Cortical Cerebral	Amyloid aggregation, hyperphosphorylated tau protein, and endosome abnormalities Aβ and p-tau protein aggregates, the elevated degree of cellular apoptosis	Partial AD-like pathologies	Lack of Vasculature and microglia formation	Gonzalez et al., 2018 Raja et al., 2016
hiPSCs derived NESCs PD-hiPSCs with PINK1 mutation PD-hiPSCs derived from idiopathic PD PD-hiPSC with LRRK2-G2019S mutation	Midbrain Midbrain Midbrain Midbrain	Synaptic connection and electrophysiological activity Abnormal cell proliferation, differentiation, and apoptosis. Normal cell morphology but altered gene expression A decrease in the number and complexity of mDANs	Not addressed PD-like phenotypes Not addressed PD-relevant phenotypes.	Lack of Vasculature and microglia formation	Monzel et al., 2017 Jarazo et al., 2019 Chlebanowska et al., 2020 Smits et al., 2019

*HD-hiPSCs, human induced pluripotent stem cells from patients with Huntington's disease; FAD-hiPSCs, human induced pluripotent stem cells from patients with familial Alzheimer's disease; DS-hiPSCs, human induced pluripotent stem cells from individuals with Down's syndrome; PD-hiPSCs, human induced pluripotent stem cells from individuals with Parkinson's disease*.

**Alzheimer's disease** is a complex, multifactorial disorder with genetic, and environmental factors that ultimately lead to premature neuronal death. Early-onset familial AD (EOAD) is a rare autosomal dominant form of AD with predictive gene mutations, but genetic and environmental factors that affect susceptible genes are the major risk factors for late-onset AD (LOAD), which is also called sporadic AD. Furthermore, genetic variations by susceptibility genes play a crucial role in determining the risk of LOAD (Papaspyropoulos et al., 2020). Raja et al. (2016) demonstrated disease phenotypes (amyloid and tau pathology) in organoids (Table 4), which were derived from multiple familial AD (FAD) patients whose genotype consisted of amyloid precursor protein (APP) duplication or presenilin1 (*PSEN1*) mutations. Moreover, the treatment of patient-derived organoids with β- and γ-secretase inhibitors significantly reduced amyloid and tau pathology. Recently, Gonzalez et al. (2018) generated cerebral organoids from patient iPSCs affected by familial AD or Down syndrome (DS). These organoids manifested pathological properties of AD, such as Aβ and p-tau protein aggregates, as well as the elevated degree of cellular apoptosis (Table 4). These studies demonstrate that AD pathology can be at least partially recapitulated in patient-derived organoids and, compared to current methods, these organoids offer a new platform for the investigation of drug screening for therapeutic intervention.

**Parkinson's disease** is a major neurodegenerative disorder that is characterized by the loss of DA neurons within the substantia nigra pars compacta and the presence of numerous Lewy bodies in surviving neurons (Jellinger, 2009). The degeneration of these neurons causes a variety of motor dysfunctions, including tremors, rigidity, and bradykinesia, which gradually increase over time. Only 10% of PD is described as familial and involves several mutated genes, including *SNCA, PRKN, PINK1, DJ-1, LRRK2*, and *VSP35*. While the mechanisms of the neurodegenerative process in PD are still unknown, most PD patients suffer from idiopathic or sporadic forms of PD, emerging from unknown causes.

Focusing on familial forms of PD, the mutation of the α-synuclein (SNCA) gene leads to protein aggregation and is thought to be a major cause of the pathophysiology of PD (Jellinger, 2009). Over 385 iPSC lines have been developed to investigate disease-like phenotypes of PD since 2011. Among these lines, most of them are familial PD with a single-gene mutation (Tran et al., 2020). For example, patient-derived iPSCs were prepared with *PINK1* mutations and differentiated into neuroepithelial stem cells (NESCs), and later into neurons (Jarazo et al., 2019). This finding demonstrates that patient-derived NESCs can recapitulate PD-like phenotypes, such as reduction of differentiation efficiency, impaired mitophagy capacity, and increased cell death of dopaminergic neurons. However, patient-derived iPSCs demonstrate only partial disease phenotypes, such as the reduction of cell viability or neuronal differentiation defects (Table 4).

The limitations of 2D iPSCs cultures suggest that the generation of patient-derived 3D organoids are needed to provide more relevant models that more accurately recapitulate the pathophysiology of PD, as organoids can also provide more accurate models of the genetic- and epigenetic-mediated disease phenotypes. Recently, 3D ventral midbrain-like organoids have been developed that consist of dopaminergic neurons and neuromelanin granules, similar to what is observed in human substantia nigra tissue (Jo et al., 2016; Monzel et al., 2017; Qian et al., 2018). These 3D organoids faithfully mimic the early developmental and functional patterning of brain regions, providing highly accurate simulations of degenerative neuropathology (Shi et al., 2012). A very recent study indicated that treatment with 2-hydroxypropyl-β-cyclodextrin (HP-β-CD) restores differentiation of patient-specific neurons in midbrain organoids by increasing neuronal mitophagy capacity (Jarazo et al., 2019).

**Primary microcephaly and autism spectrum disorder** are neurodevelopmental disorders whose neuronal pathologies may be modeled by 3D organoids. Primary microcephaly (MCPH) is a neurodevelopmental disorder, which causes a reduction of brain size due to autosomal recessive mutations in several genes. Mutations for several of the known genes in mice have failed to recapitulate MCPH pathogenesis, but recent evidence shows that recapitulation of such pathogenesis of MCPH can be achieved using patient-derived iPSCs and cerebral-organoid cultures (Lancaster et al., 2013; Li et al., 2017). For example, patient-derived organoids exhibit premature neural differentiation at the expense of early neural progenitors, disrupted radial glia spindle orientation, reduced total neural tissue, and increased neuronal outgrowth (Table 5).

**Table 5:** Modeling neurodevelopmental disorders in hiPSC-derived organoids.

**Cell types**	**Organoid types**	**Characterizations**	**Recapitulation of disease phenotypes**	**Limitations**	**References**
hiPSCs derived from MCPH patients with Aspm mutation	Cerebral	A decreased size of EBs. Anormal cell aggregates, self-organizing, and defective layer lamination. Fewer mature neurons and defective neuronal activities	MCPH like phenotypes	Lack of Vasculature and microglia formation	Lancaster et al., 2013; Li et al., 2017
hiPSCs derived from severe idiopathic ASD	Cerebral	Accelerated cell cycle and overproduction of GABAergic inhibitory neurons.	ASD-relevant phenotypes		Mariani et al., 2015
hiPSCs derived from ASD patients	Cerebral	Abnormal neurogenesis and reduced synaptogenesis leading to functional defects in neuronal networks.	ASD-relevant phenotypes		Marchetto et al., 2017
hiPSCs with CHD8 mutation	Cerebral	Alteration ASD and SZ candidate genes expression	Not addressed		Wang et al., 2017

*hiPSCs, human induced pluripotent stem cells; MCPH, microencephaly; ASD, autism spectrum disorder; EBs, embryoid bodies; GABAergic, relating to gama aminobutyric acid; SZ, schizophrenia*.

Autism spectrum disorders (ASDs) consist of a spectrum of neurodevelopmental disorders and are characterized by impaired social interaction, repetitive or restrictive behaviors, and problems with speech. The genetic and environmental risk factors have been shown to contribute to ASD prevalence. Recently, 2D- and 3D- cultures of hiPSCs, which were derived from patients with idiopathic autism (with an increased head/brain size), recapitulated the phenotypic signature of the autistic brain, such as abnormal cell proliferation and an over-production of inhibitory neurons due to dysregulation of the transcriptional cascade (Mariani et al., 2015; Marchetto et al., 2017). The development of 3D organoids for ASD promises to elucidate some of the neuronal mechanisms of this disorder, as well as providing useful insights for therapeutic interventions.

### Recapitulation of Disease Phenotypes Associated With the Epigenetic Signatures in Patient-Derived Organoids

Epigenetic alterations can regulate the expression of genes through chromatin remodeling without changing its DNA sequence. Our DNA code remains fixed for life and all parts of our body, such as cell types and organs, are characterized by one genome, but the epigenome can be organ-, or even, cell-specific. DNA methylation, modifications of core histones, and non-coding RNA (ncRNA) are major epigenetic mechanisms that act jointly in chromatin remodeling for suppression or induction of gene expression. These epigenetic mechanisms are dynamic and cell-specific; they display inter-individual variability and can also occur in non-dividing cells, such as neurons (Fraga et al., 2005). Nutrients, pollutants, chemicals, physical and mental stress are major environmental factors, which can alter epigenetic markers in the developing and adult organism.

Epigenetic alterations are also associated with aging. For example, global DNA methylation levels are decreased with aging (Fraga et al., 2005). In addition, gene mutation can cause secondary epigenetic modifications (Cakir et al., 2020). Thus, the epigenetic alterations have been implicated in a diverse range of cellular functions and pathologies, which are generally associated with a repressed chromatin state and inhibition of promoter activity, such as transcriptional repression (Navarro-Sánchez et al., 2018). For example, cell proliferation and differentiation during prenatal development are regulated by epigenetic mechanisms.

The genetic and epigenetic variations in patient-derived iPSCs change their differentiation efficiency and developmental capacity (Liang and Zhang, 2013). A major concern for disease modeling is that the genetic and epigenetic variations detected in iPSCs may cause unexpected phenotypic changes after the iPSCs differentiate into target cells. In addition, such variations in hiPSCs may lead to the acquisition of phenotypes that are unrelated to the disease being modeled or to the disappearance of disease-related phenotypes (Liang and Zhang, 2013). Studies show that cell reprogramming, such as genes that change fibroblasts into iPSCs, removes a significant epigenetic modification (Perrera and Martello, 2019). Therefore, the biggest challenge for using iPSCs from patients is to recapitulate the pathogenesis of the neurodevelopmental and neurodegenerative diseases that are induced by environmental factors, while epigenetic modifications are partially recapitulated following neuronal maturation *in vitro* (Parr et al., 2017).

Genetic predisposition and epigenetic alterations are both considered to contribute to neurodegenerative diseases, including AD and PD. Although iPSC-derived *in vitro* models cannot display the full range of defects that contribute to neurodegenerative diseases, they are useful for modeling cellular and molecular abnormalities of neuronal development and contribute to our understanding of the causes and progression of neurodegenerative diseases (Lewis and Kroll, 2018). However, the successful use of hiPSCs to model neurodegenerative disease requires assuring genetic and epigenetic stability.

Genome-wide studies demonstrated several point mutations in all hiPSC, which raise concerns over their ability to model disease states, as well as their safety for clinical applications (Perrera and Martello, 2019). Moreover, the additional mutations in hiPSCs during the several passages that occur when cells are cultured for long periods of time (Liang and Zhang, 2013) may result in increased susceptibility to diseases or abnormal cell morphology and functions.

Recently, the data from transcriptome profiles and epigenome-wide sequencing of cerebral organoids, when compared with human fetal brain (Luo et al., 2016), revealed a recapitulation of the key characteristics of human brain development, including regional cell specification, the formation of progenitor layers, and the generation of diverse types of functional neurons (Lancaster et al., 2013). Importantly, the transcriptomic dynamics were recapitulated in hiPSC-derived organoids, which were equivalent to fetal brain of gestational week 8–16 (Luo et al., 2016). Luo et al. (2016) also demonstrated a new type of cytosine DNA methylation in non-CG contexts (mCH) that indicate transcriptional repression in later brain development (Luo et al., 2016).

However, the fate of patient-derived organoids is found to be dependent on genetic inheritance and disease phenotype of its constituent hiPSCs, which often require exogenous exposure of disease-inducing factors for the pathogenesis of diseases to develop. This indicates that disease phenotypes are associated with genetic mutations and environmental factors. If cell reprogramming removes the epigenetic code and cell aging is associated with sporadic neurodegenerative diseases, like PD and AD, then the accurate recapitulation of stable disease-like phenotypes in patient-derived organoids is a major challenge that will need to be addressed.

## Conclusions and Perspectives

Patient-derived brain organoids have provided new insights into disease modeling and have opened new possibilities for personalized medicine. Even though 3D cultures of patient-derived iPSCs hold great promise for modeling diseases, such as AD, PD, and HD, only a few studies have effectively utilized 3D vascularized organoids. New and advanced methods are required to overcome the current limitations for creating functional vascular systems and for accurately recapitulating pathogenesis and many complex physiological features of the human brain. In addition, batch-to-batch variations are often observed among organoids, due to genetic and epigenetic variations resulting from the origin or reprogramming approaches used to obtain the hiPSCs, which may change the differentiation and function of the organoids from which they were derived. Therefore, a better understanding of cell-ECM and cell-cell interaction, incorporation of microglia and vascularization into the models, and the use of self-patterning, and advanced protocols for iPSC reprogramming are required to recreate reliable 3D organoid models. Such vascularized organoids might have diverse applications as BBB model systems for various diseases, including neurodegenerative and neurodevelopmental disorders, as well as for transplantation to treat brain injury. Moreover, they can also be used as models for drug-screening, brain-infection, and neurotoxicity studies.

Although hiPSC-derived organoid models are promising tools to recapitulate developmental and disease phenotypes, these *in vitro* models are not a substitute for animal models, especially those that model behavior and psychological disorders. In addition, animal models are still needed to investigate long-term drug responses and multi-organ drug toxicity tests. However, because many promising pharmacological findings from animal studies have high failure rates in clinical trials, the parallel use of hiPSC-derived organoids could augment the translatability of animal studies to humans. As hiPSC-derived *in vitro* models more closely resemble complex *in vivo* processes and the current limitations of organoids are addressed, a new era of translatable therapies will emerge. Although there is still a long way to go before iPSC-based methods can be used directly in clinics, the modification of current organoid technology with genome editing, drug screening, and other technologies, as well as precise characterization of these organoids using cutting edge technologies, will certainly advance the generation of new therapies for human developmental and neurodegenerative diseases.

## Author Contributions

RB had the main idea of the article, made a substantial contribution in the literature survey, designed and written the original manuscript, designed original figures and tables, corrections, and critical revisions. SB contributed to the revision, modification of the manuscript, and plotting of the figure. GD reviewed and edited the manuscript including figures and tables. All authors contributed to the article and approved the submitted version.

## Conflict of Interest

The authors declare that the research was conducted in the absence of any commercial or financial relationships that could be construed as a potential conflict of interest.
